# Social reward responsiveness as a moderator of the association between perceived bonding with infants and depressive symptoms in postpartum women

**DOI:** 10.3758/s13415-025-01286-0

**Published:** 2025-03-18

**Authors:** Emilia F. Cárdenas, Maya Jackson, Julia Garon-Bissonnette, Kathryn L. Humphreys, Autumn Kujawa

**Affiliations:** Department of Psychology and Human Development, Vanderbilt University, Nashville, TN 37203 USA

**Keywords:** Depression, Bonding, Reward processing, Postpartum

## Abstract

There is a need to identify neurobiological and psychosocial risk processes for postpartum depression (PPD). Previous research links low reward responsiveness with lower reported affiliation or bond to one’s infant and PPD symptoms, but the potential moderating role of reward processing in the relationship between bonding with infants and PPD has yet to be examined. The current study (*n* = 117) used a personally salient social reward task to examine whether neural reward responsiveness moderates the association between bonding difficulties and PPD symptoms. Postpartum women (*n* = 93) completed the Postpartum Bonding Questionnaire biweekly following childbirth until 8 weeks postpartum, with responses averaged across timepoints. At 8 weeks postpartum, participants completed an electroencephalogram (EEG) Social Incentive Delay task, which included social reward feedback indicating participants would see a personally salient social reward (i.e., cute photo of their infant) and neutral feedback indicating participants would see a neutral image while electroencephalogram data were collected. Participants also self-reported depressive symptoms. A larger social RewP was associated with greater perceived bonding difficulties, and social RewP and self-reported bonding interacted to predict PPD symptoms. The association between bonding difficulties and greater PPD symptoms was statistically significant only for women low in social reward responsiveness. RewP to personally salient infant social reward may be a relevant measure of brain function in the context of maternal perceived bonding and PPD risk.

## Introduction

Postpartum depression (PPD) is a growing global concern, with prevalence rates for PPD reaching 10% to 20% worldwide (Wang et al., 2021). Postpartum depression develops within 1 year of childbirth and can encompass a range of symptoms (e.g., unpredictable mood fluctuations and intrusive thoughts that may relate to harming oneself or one’s infant; Modak et al., 2023). In addition to affecting birthing parents (hereafter: mothers), PPD symptoms are associated with short- and long-term impairments in infants’ functioning, including feeding problems, emotional difficulties, and lower cognitive functioning (Fitelson et al., 2010). Given the rate of PPD and potential consequences for both mothers and infants, there is a clear need to better understand risk processes associated with depressive symptoms in the postpartum period.

One potential factor associated with PPD symptoms is mothers’ difficulties in feeling bonded with infants (Tichelman et al., 2019). These feelings of bond to one’s infant are typically measured with self-report questionnaires (Brockington et al., 2001), and concurrent associations between postpartum PPD symptoms and bonding difficulties have been previously observed (Kerstis et al., 2016; Tietz et al., 2014). These associations may partly reflect shared attributes between mothers’ symptoms and perceptions. Consistent with this, negative perceptions represent one core symptom of postpartum depressive episodes (e.g., see Beck’s cognitive theory of depression; Beck, 1967), such that the direction of the association between bonding difficulties and PPD symptoms has often been conceptualized as reciprocal or bidirectional. However, recent evidence from two studies suggests that perceptions of difficulties in bonding may temporally precede and precipitate PPD symptoms (Hiraoka et al., 2024; Ohara et al., 2017). Specifically, in a sample of 433 women followed from pregnancy through 18 months postpartum, Hiraoka et al. (2024) found that mothers’ reported bond predicted subsequent PPD symptoms. However, PPD symptoms did not subsequently predict mothers’ reported bond. As such, difficulties bonding with one’s infant may be a risk factor underlying the development of PPD symptoms, raising questions about other factors that moderate these associations to promote resilience or increase risk.

The transition to parenthood is a time in which infant cues are thought to be particularly salient and rewarding. Postpartum women exhibit heightened reward reactivity to infant cues at the neural level (Ranote et al., 2004), which is thought to underlie the establishment and maintenance of caregiving behaviors (e.g., attention, empathy) as well as mothers’ feelings of affiliation with or bond toward their infant (Swain et al., 2007). In the postpartum period, new mothers have been found to display heightened brain activation in reward regions (e.g., ventral striatum, dorsal striatum, medial prefrontal cortex) when listening to the sound of an infant crying relative to both white noise and silence (Pechtel et al., 2013) and when viewing images of their own infant versus an unknown child (Stoeckel et al., 2014). Such activation of reward circuits is hypothesized to play a role in feelings of connection and affiliation. In the context of the postpartum period, the extent to which mothers feel a bond with their infants as rewarding may impact risk for PPD symptoms. Consistent with this possibility, there is evidence that mothers with depression exhibit relatively reduced reward-related neural responses to stimuli associated with their infants specifically (Post & Leuner, 2019).

Growing evidence supports the use of neuroscience methods, such as event-related potentials (ERPs), as tools for examining alterations in reward processing in depression (Kujawa, 2024). Event-related potentials are safe, economical, and comfortable tools for measuring neural activity across the peripartum period, including neural reward responsiveness (Maupin et al., 2015). The reward positivity (RewP), over frontocentral sites of the scalp, is an ERP measuring neural reward responsiveness that emerges 250–350 ms after receipt of reward compared with loss or neutral feedback (Proudfit et al., 2015). Neural reward responsiveness is commonly assessed in relation to monetary rewards (Mulligan et al., 2019), and there has been growing interest in extension to social domains (Pegg et al., 2019). Social RewP has typically been elicited in tasks where participants are led to believe they are receiving feedback from peers (e.g., likes vs. dislikes; Kujawa, 2024; Wang et al., 2021). The current study is unique in that it is among the first to adapt an established monetary reward task using personally relevant, salient social rewards for new mothers (i.e., cues indicating they will see a cute photo of their own infant). Furthermore, the RewP has been associated with indicators of bonding and depressive symptoms, suggesting that it may be relevant to understanding the emergence of PPD symptoms in the transition to parenthood. Specifically, prospective associations have been observed between the RewP in pregnancy and self-reported mother-to-fetus investment approximately 1 year postpartum (Mulligan et al., 2021). In addition, in a sample of pregnant women, a reduced RewP to monetary rewards was associated with greater antenatal depressive symptoms (Mulligan et al., 2019). Low reward responsiveness also exhibits moderate effects of stress on depression outside of the peripartum period (Pegg et al., 2019). Women with preexisting tendencies to experience low reward responsiveness, particularly in the social domain, may also experience more difficulty in the transition to parenting when difficulties also exist in the mother-infant bonds (Admon & Pizzagalli, 2015; O’Dea et al., 2023).

The goal of the current study was to leverage a personally salient social reward task to examine associations between self-reported bonding difficulties, social RewP, and PPD symptoms. Reward-related brain function (i.e., RewP) has been identified as a potential predictor of bonding and depressive symptoms during the transition to parenthood (Mulligan et al., 2019). Previous research has identified some potential moderators of associations between perceived bonding and PPD (e.g., oxytocin; perceived parental stress). Given previous findings regarding the role of reward in the context of neural responses to infant cues and mother’s reported bond with her infant in the postpartum period, social RewP may be a moderator for the associations between perceived bonding and PPD (Gholampour et al., 2020; Mulligan et al., 2021). In the current study, postpartum women completed the Postpartum Bonding Questionnaire biweekly following childbirth until 8 weeks postpartum, with participants responses averaged across timepoints. Building on the growing use of social versus monetary reward, we decided to develop a novel, personally tailored social reward task that utilized photos of participants' infants. At the 8-week postpartum session, participants completed a Social Incentive Delay task, which included social reward feedback indicating participants would see a personally salient social reward (i.e., photo of their infant) and neutral feedback indicating participants would see a boring image while electroencephalogram data were collected. Analyses focused on the RewP component—an index of reward processing. At 8 weeks postpartum, participants completed a self-report measure of depressive symptoms. Given that prenatal depressive symptoms are one of the strongest predictors of postpartum depressive symptoms (Gaillard et al., 2014; Pfost et al., 1990), we included prenatal depressive symptoms as a covariate in all analyses. Our first goal was to test whether perceived bonding is related to PPD symptoms and social RewP. We hypothesized that perceived bonding difficulties would be positively associated with PPD symptoms and negatively associated with social RewP. Our second goal was to test whether individual differences in social RewP moderate the association between perceived bonding and PPD symptoms. We further hypothesized that the association between perceived bonding difficulties in mothers and PPD symptoms would be greater for mothers with relatively blunted social RewP amplitude to images of their infant compared with mothers with relatively enhanced social RewP amplitude to images of their infant.

## Methods

### Participants

Pregnant individuals were recruited to participate in a study assessing predictors of postpartum depression. Eligibility criteria for the longitudinal study included (a) pregnant and approximately 20 weeks gestation at the time of enrollment; (b) age between 18 and 40 years; (c) no previous diagnosis of mania/bipolar disorder, psychosis, or borderline personality disorders; and (d) fluency in English (Table 1). Eligibility criteria also preclude women pregnant with multiples or a fetus with known congenital issues. In total, 120 participants (*M*_*age*_ = 31.09 years, *SD* = 4.81) from the greater Nashville metropolitan area were enrolled in the study around 20 weeks gestation. Although not all pregnant individuals identify as a woman, 100% of participants in this sample endorsed woman as their gender identity. Participants were recruited through clinics in the community, online advertisements, flyers, and community events. Participants were enrolled as close as possible to 20 weeks gestation and were reassessed at approximately 34 weeks gestation and 8 weeks postpartum. Following delivery, participants completed a biweekly perceived postpartum bonding questionnaire through a mobile application beginning the week following childbirth until 8 weeks postpartum. Of the 120 participants enrolled, 117 participants completed a prenatal measure of depressive symptoms, 93 completed all four postpartum bonding questionnaires, and 91 completed the postpartum EEG session.

**Table 1:** Descriptive statistics of participant demographics

Variable	Percentage
Race	
White	79%
Black and/or African American	10%
Multi-racial or other race	8%
Asian	3%
Ethnicity	
Non-Hispanic	90%
Hispanic	10%
Partnership	
Married or in a domestic partnership	85%
Single and never married	12%
Divorced	3%
Employment status	
Employed for wages	85%
Homemakers	6%
Out of working and looking for work	3%
Student	3%
Self-employed	2%
Other	1%
Annual household income	
$0–5,000	1%
$5,001–15,000	3%
$15,001–30,000	5%
$30,001–60,000	17%
$60,001–90,000	22%
$90,001–150,000	30%
Greater than $150,000	22%
No. prior children	
None	65%
One child	26%
Two children	5%
Three children	1%
Four children	3%

### Procedures

Participants first completed a phone screen to determine eligibility. Then, they completed a battery of computer tasks during continuous collection of EEG during two gestational sessions and one postpartum session (Cárdenas et al., 2023). At both the second (i.e., approximately 20 weeks gestation) and third (i.e., 34 weeks gestation) trimesters, participants completed self-reported measures of depressive symptoms and demographic background. Participants were emailed survey links to complete the Postpartum Bonding Questionnaire in REDCap every other week following childbirth until the 8-week postpartum assessment to assess self-reported bonding difficulties. In addition to the battery of EEG computer tasks completed during gestation, participants completed an additional computer task, the Social Incentive Delay task, at their 8-week postpartum assessment. The Social Incentive Delay task was only administered at the 8-week postpartum assessment, because this task required photos of the participants’ infant. We have previously published EEG data during pregnancy (Cárdenas et al., 2023), and analyses for the current study focus on the third EEG postpartum and postpartum questionnaires.

### Task and measures

#### Maternal-reported bonding difficulties

Participants completed the Postpartum Bonding Questionnaire (PBQ) to assess maternal feelings of bonding with their infant (Brockington et al., 2001). Specifically, the PBQ is a self-reported measure assessing difficulties, or impairments, in caregivers’ perception of bonding with their infant. The PBQ has 25 items rated on a 6-point Likert scale from 0 (*Always*) to 5 (*Never*). For this measure, participants were asked to indicate how often statements about bonding were true. Whereas 17 items were negatively worded (e.g., The baby does not seem to be mine), 8 items were positively worded (e.g., I feel close to my baby); those were reversed scored to reflect difficulties. In this study, we used the general factor of impaired bonding within the PBQ (comprising 12 items), with higher scores indicating increased difficulties in feeling bonded with their infant. Internal consistency (Cronbach’s alpha) ranged from 0.74 and 0.83 across timepoints. Participants’ biweekly PBQ scores across the first 8 weeks postpartum were averaged to provide a measure of average maternal feelings of bonding with their infant across the first 8 weeks postpartum.

#### Personally salient social incentive delay task

**Fig. 1:**
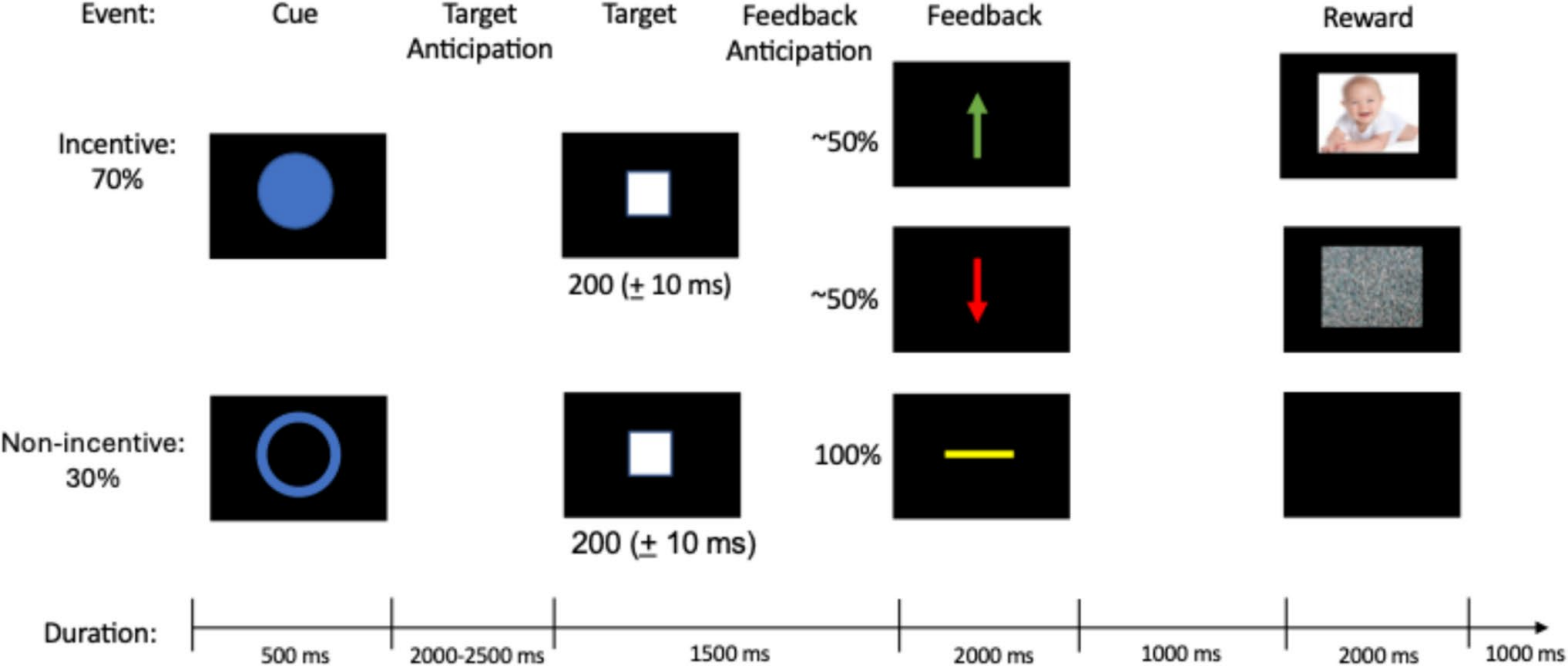
Sample trial on the social incentive delay task

This novel Social Incentive Delay task (Fig. 1) is a participant-specific social reward task that was developed for this project and adapted from the ERP version of the Monetary Incentive Delay task (Novak & Foti, 2015). At the start of each trial, participants were presented with a cue that indicated either a social incentive trial (i.e., blue circle) or a nonincentive trial (i.e., blue outline of a circle) for 500 ms. Following the cue, and as an anticipatory period, a fixation cross (i.e., white plus sign) was presented for 2,000–2,500 ms. Then, a white box was presented as a target. Participants were instructed to respond to the target by pressing the left mouse button as quickly as possible. Target presentation time was dynamically adjusted to achieve an approximately 50% success rate. Target presentation began at 200 ms and decreased by 100 ms following successful responses and increased by 10 ms following unsuccessful responses. Following target presentation, a fixation cross was presented for a total of 1,500 ms from target presentation to feedback onset. On incentive trials, a green upward arrow appeared for responses to the target within the presentation period, indicating reward feedback. The green upward arrow was then followed by a fixation cross for 1,000 ms followed by a photograph of their infant for 2,000 ms. A red downward arrow appeared for responses outside of the presentation period, indicating neutral feedback. The red downward arrow was followed by a fixation cross for 1,000 ms followed by a photograph of rocks for 2,000 ms. We used photographs of rocks because previous literature used rocks as neutral, nonreward, and not motivationally salient stimuli (Mühlberger et al., 2017). On nonincentive trials, a yellow dash was presented, indicating that the participants would not receive performance feedback or see a photo, regardless of reaction time. The yellow dash was followed by a fixation cross for 1,000 ms and then followed by a blank screen for 2,000 ms. A fixation cross for 1,000 ms was presented prior to the start of the next trial. The task provided a sufficient number of trials to reliably elicit the RewP (Ethridge & Weinberg, 2018). Analyses focused on the neural response to reward vs. negative feedback (i.e., green and red arrows).

#### Prenatal and postpartum depressive measures

Participants completed the Inventory of Depression and Anxiety Symptoms (IDAS), a validated measure of recent (i.e., past 2 weeks) depressive and anxiety symptoms at both the prenatal and postpartum sessions (Watson et al., 2007). The IDAS produces several subscales, including the 20-item general depression scale. For the general depression scale, scores above 55.5 (of 100) indicate possible major depressive disorders (Stasik-O’Brien et al., 2019). Internal consistency (Cronbach’s alpha) ranged from 0.87 to 0.92 across assessments.

The Diagnostic Interview for Anxiety, Mood, and Obsessive–Compulsive and Related Neuropsychiatric Disorder (DIAMOND; Tolin et al., 2018), a semistructured interview of DSM-5 disorders, was administered by research staff at each assessment to characterize clinical diagnoses for descriptive purposes. Diagnoses were reviewed during weekly clinical supervision and verified by a licensed clinical psychologist. A sample of 19 DIAMOND interviews at the first session was reviewed by an independent rater for reliability of current major depressive disorder (MDD; Cohen’s kappa = 1) and persistent depressive disorder (PDD; Cohen’s kappa = 1), indicating perfect agreement between the raters (McHugh, 2012). At the first interview session, 52% met criteria for a past or current MDD episode and 14% met criteria for a past or current PDD episode. Participants were interviewed again in late pregnancy and approximately 8 weeks postpartum with 13% endorsing MDD or PPD episode at one or both timepoints.

### Data analytic approach

#### EEG data acquisition and processing

**Fig. 2:**
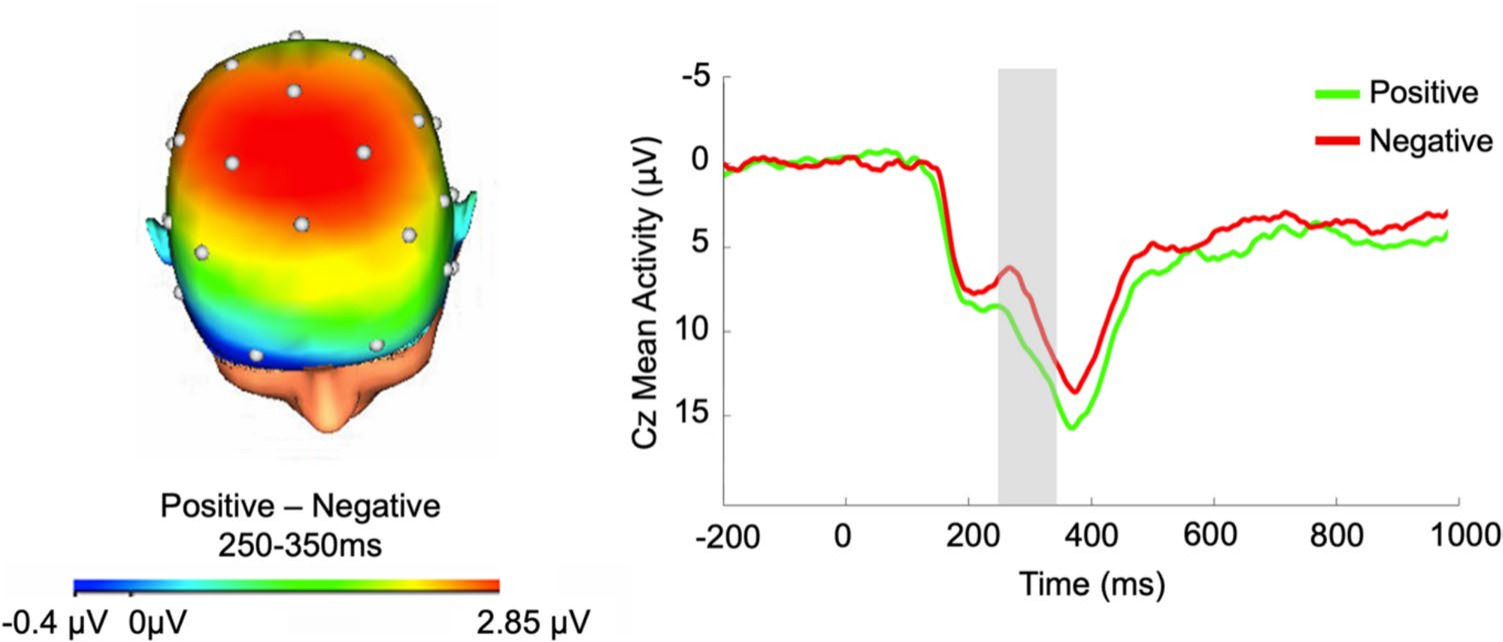
Scalp topography and grand averaged social RewP waveform to social reward feedback minus responses to neutral feedback at Cz 250–350 ms after feedback onset

EEG data was recorded with a 32-channel BrainProducts actiCHamp system (Munich, Germany) using a standard 10/20 layout. A 32-channel cap was used for all participants, but at the beginning of the study, we focused only on 16 channels in applying gel and lowering impedances to reduce setup time, consistent with recommendations to minimize time in close contact early in the COVID-19 pandemic before vaccines were available (Simmons & Luck, 2020). There was no statistically significant difference in the RewP unstandardized residual between participants with 32 (*n* = 75) vs. 16 (*n* = 7) electrodes (Cohen’s *d* = 0.38, *p* = 0.341). BrainVision Analyzer software (Munich, Germany) was used to process the EEG data. Data was processed in BrainVision Analyzer (BVA) using best practices (Luck et al., 2000). Impedances were reduced to 30 ﻿kΩ. A 24-bit resolution and sample rate of 1,000 Hz were used to digitize the recordings. Data was referenced to mastoid electrodes and band-pass filtered with 0.1 and 30 Hz as cutoffs. Data was segmented − 500 ms before and 1,000 ms after stimuli presentation. Ocular correction was conducted with a modification of Gratton’s algorithm (Gratton et al., 1983). Vertical (VEO) and horizontal (HEO) electrooculogram were monitored bipolarly from sites above and below, as well as beside the outer canthus of participants’ eyes. Owing to modified COVID protocols, we did not apply facial electrodes for the participants at the beginning of the study. For these participants, we used FP1 in lieu of VEO and FT9 and FT10 in lieu of HEO. There was also no statistically significant difference in the RewP unstandardized residual between participants in which we used HEO and VEO (*n* = 53) vs. FP1 and FT9/FT10 (*n* = 29) for ocular correction (Cohen’s *d* = 0.18, *p* = 0.440). Automatic artifact rejection criteria was a voltage step greater than 50 ﻿µV between sample points. The maximum voltage difference between points was capped at 175 ﻿µV for trials and the minimum voltage difference parameters was 0.5 ﻿µV within 100-ms intervals. After automatic artifact rejection, data were inspected visually to reject any remaining artifacts. Segmented data were averaged across each condition, and baseline corrected 200 ms to 0 ms prior to feedback onset. Analyses focused on the RewP component, scored between 250–350 ms at Cz consistent with prior research (Pegg et al., 2019) and grand averages in this sample (Fig. 2).

For the social incentive delay task, eight participants were excluded for poor data quality (e.g., low available segments after artifact rejection) and one participant was excluded because of technical issues. There was available social incentive delay task EEG data for 82 participants. Following the artifact rejection procedures, included participants had on average 23.52 segments (*SD* = 2.54, range = 17–28) for the social reward feedback condition and on average 24.65 segments (*SD* = 3.02, range = 15–33) for the neutral feedback condition. The RewP split-half reliability was excellent for both the RewP to social reward feedback (Spearman-Brown coefficient: *r*_*SB*_ = 0.89) and neutral feedback (Spearman-Brown coefficient: *r*_*SB*_ = 0.87). The RewP was significantly larger for positive (*M* = 10.93; *SD* = 5.97) compared with neutral (*m* = 8.36, *SD* = 5.59) feedback: *t*(81) = 5.49, *p* < 0.001; Cohen’s *d* = 0.61). Furthermore, reaction time was significantly faster for incentive (*m* = 222.85, *SD* = 28.36) compared with nonincentive (*m* = 264.68, *SD* = 57.66): *t*(86) =  − 9.42, *p* < 0.001; Cohen’s *d* =  − 1.01).^1^ We calculated the unstandardized residual to the RewP for social reward feedback, adjusting for the RewP to neutral feedback (Meyer et al., 2017).

#### Data analyses

Descriptive statistics (i.e., means, standard deviations, minimums, maximums, ranges) were calculated for all key study variables to inspect distribution and quality of data. Bivariate correlations were conducted to examine associations between study variables, with a particular focus on associations between bonding difficulties, PPD symptoms, and social RewP. We then conducted Little’s missing completely at random (MCAR) test, which was null (*p* = 0.596). Because this test showed that the data were missing at random, we used full information maximum likelihood (FIML) in main analyses (regressions) via the lavaan package (Rosseel, 2012) in R version 2022.02.2 (R Core Team, 2021).

To test social reward responsiveness as a moderator of associations between bonding difficulties and PPD symptoms, we used multiple regression analyses with an interaction term to model the effect of bonding difficulties (independent variable) on PPD symptoms (dependent variable) at different levels of social reward responsiveness (moderating variable). Analyses focused on the unstandardized residual to the RewP for social reward feedback adjusting for the RewP to neutral feedback. We included prenatal depressive symptoms as a covariate. Specifically, we included an average of the participants’ depressive symptoms at the second and third trimesters of pregnancy. In cases of attrition before the third trimester of pregnancy, we only used depressive symptoms in analyses from the second trimester of pregnancy. We also tested this model without depressive symptoms as a covariate. Furthermore, we tested this model covarying EEG methods differences to test how robust results are to different scoring and analysis decisions.

## Results

Table 2 presents descriptive statistics for all study variables. Table 3 presents bivariate correlations among primary variables. Higher prenatal depressive symptoms were associated with increased PPD symptoms as well as greater perceived bonding difficulties. There was a nonstatistically significant trend (*p* = 0.051; small effect size) between greater postpartum depressive symptoms and greater perceived bonding difficulties. Contrary to our hypothesis, heightened social RewP was associated with greater perceived bonding difficulties. There was no statistically significant association between social RewP and prenatal or postpartum depressive symptoms.

**Table 2:** Descriptive statistics of study variables

Variable	n	Mean	SD	Min	Max	Range
Prenatal depressive symptoms	117	37.12	10.78	22.00	92.00	70.00
Postpartum depressive symptoms	94	36.59	8.64	23.00	65.00	42.00
Perceived bonding difficulties	93	4.42	3.62	0.00	20.75	20.75
Social RewP	82	0.00	4.06	− 10.45	10.57	21.03

Prenatal depressive symptoms = averaged second trimester and third trimester depressive symptoms or just second trimester depressive symptoms for participants with missing data at third trimester. Social RewP UR = unstandardized residual of the response to social reward feedback accounting for responses to neural feedback

**Table 3:** Bivariate correlations among study variables

Variable	1	2	3	4
1. Prenatal depressive symptoms	-			
2. Postpartum depressive symptoms	**0.62	-		
3. Perceived bonding difficulties	*0.24	0.21	-	
4. Social RewP	0.12	0.22	*0.25	-

*Correlation was significant at the 0.05 level (2-tailed). **Correlation was significant at the .01 level (2-tailed). Prenatal depressive symptoms = averaged second trimester and third trimester depressive symptoms or just second trimester depressive symptoms for participants with missing data at third trimester. Social RewP = unstandardized residual of the response to social reward feedback accounting for responses to neutral feedback. The correlation between perceived bonding difficulties and postpartum depressive symptoms were n.s. (*p* = .051; small effect)

**Fig. 3:**
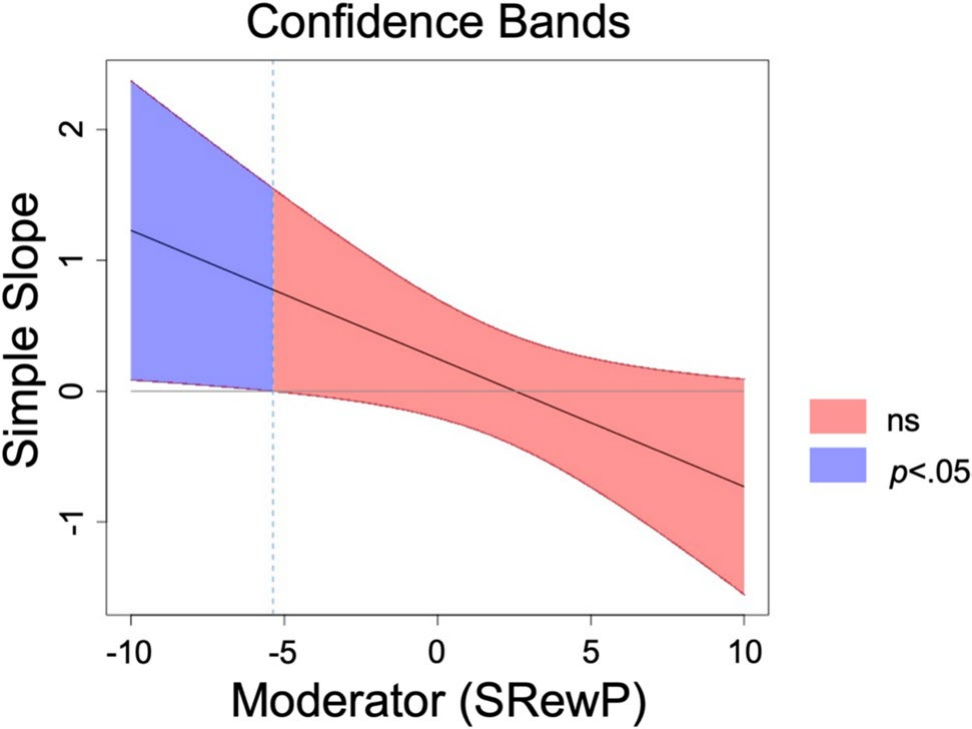
Associations between perceived bonding difficulties and postpartum depressive symptoms as a function of social RewP. Note. Social RewP = unstandardized residual response to social reward feedback accounting for responses to neutral feedback. Ns = not significant. The figure displays the simple slope of the association between the focal predictor (i.e., perceived bonding difficulties) and outcome (i.e., PPD symptoms) as a function of different levels of the moderator (social RewP). At low levels of social RewP (< − 5.32), bonding difficulties are positively associated with PPD symptoms, whereas the association is smaller and does not reach statistical significance at moderate to high levels of social RewP

A multiple regression model was tested to investigate whether the association between perceived bonding difficulties and PPD symptoms was moderated by social RewP. The perceived bonding difficulties X social RewP interaction effect on PPD symptoms was statistically significant (Table 4). The region of significance for the association between the focal predictor (i.e., bonding difficulties) and outcome (i.e., PPD symptoms) showed that greater bonding difficulties were associated with greater PPD symptoms for women who showed relatively low social RewP (residual less than − 5.32 µV; Fig. 3) in anticipation of a personally relevant social reward (i.e., cute photo of their own infant). Bonding difficulties showed a trend positive association with PPD symptoms for woman who had relatively low (i.e., 16th percentile, *b* = 0.65, *SE* = 0.34, *p* = 0.062), but not average (i.e., 50th percentile, *b* = 0.25, *SE* = 0.23, *p* = 0.277) or enhanced social RewP (i.e., 84th percentile, *b* =  − 0.15, *SE* = 0.23, *p* = 0.517). When not accounting for prenatal symptoms, the interaction showed a similar effect size but did not reach significance (*b* =  − 0.11, *SE* = 0.06, *p* = 0.073, *CI* [− 0.22, 0.01]). Furthermore, the interaction effect size remained largely unchanged when covarying for EEG methods differences (32 vs. 16; *b* =  − 0.09, *SE* = 0.05, *p* = 0.061, *CI* [− 0.19, < 0.01]) or EOG electrodes (VEO and HEO vs. other method; *b* =  − 0.10, *SE* = 0.05, *p* = 0.037, *CI* [− 0.20, − 0.01]).

**Table 4:** Main and interactive effects of perceived bonding difficulties and social RewP on postpartum depressive symptoms

Variable	*b*	SE	b	*p*	95% CI	
					*LL*	*UL*
Prenatal depressive symptoms	0.52	0.09	0.60	< .001	0.35	0.68
Perceived bonding difficulties	0.25	0.23	0.10	.273	− 0.20	0.70
Social RewP	0.82	0.33	0.36	.012	0.18	1.46
Social RewP X Perceived bonding difficulties	− 0.10	0.05	− 0.30	.046	− 0.19	< − 0.01

Social RewP = unstandardized residual of the response to social reward feedback accounting for responses to neutral feedback

## Discussion

We examined the relationship between self-reported bonding difficulties, neural social reward responsiveness (i.e., RewP to social reward vs. neutral feedback), and PPD symptoms. Our first goal was to test whether bonding difficulties are associated with prenatal and postpartum depressive symptoms as well as social RewP. At the bivariate level, we found a nonsignificant trend between greater bonding difficulties and PPD symptoms and, contrary to our hypothesis, a positive association relation between heightened social RewP and greater perceived bonding difficulties. We also found no statistically significant relationship between social RewP with prenatal depression or PPD symptoms. Our second goal was to test whether bonding difficulties and social RewP were associated with PPD symptoms over and above prenatal symptoms. After adjusting for symptoms of depression in the prenatal period, we found that social RewP moderated the association between perceived bonding difficulties and PPD symptoms: perceived bonding difficulties were associated with greater PPD symptoms only for those with relatively blunted social RewP to a cue indicating they won the opportunity to see their infants’ pictures.

Our findings of the associations between perceived bonding difficulties and greater PPD symptoms for those with relatively blunted social RewP align with research linking blunted reward responsiveness with both depressive symptoms (Pegg et al., 2019) and bonding difficulties (Mulligan et al., 2021), as well as research on the connection between bonding difficulty and PPD symptoms (Kerstis et al., 2016; Tietz et al., 2014). Our results specifically suggest individuals perceiving difficulties in bonding with their infant as well as a blunted reward responsiveness to social cues may be at greater risk of PPD symptoms than individuals reporting either risk factor independently. Mothers showing low social reward responsiveness in the context of infant cues may have preexisting tendencies to experience fewer positive emotions. When paired with the added stress of perceived difficulty in bonding with their infants, these mothers may tend to further disengage and be at increased depression risk.

Although results of the moderation analysis were consistent with our hypothesis, the direct association between greater social RewP amplitude and greater perceived bonding difficulties was unexpected. This is particularly surprising given prior research indicating that mothers’ antenatal RewP was positively associated specifically with pleasure in being in proximity with their infant at around 1 year postnatal (Mulligan et al., 2021). This difference in results could be attributable to differences in measures of bonding (i.e., Postpartum Bonding Questionnaire (PBQ) in the current study and Maternal Postnatal Attachment Scale (MPAS) in Mulligan et al., 2021). The results in Mulligan et al. (2021) were specific to positive aspects of mothers’ feelings of bonding, (i.e., pleasure in proximity), which is distinct from the PBQ which is designed to identify difficulties or disturbances in postpartum bonding. As such, the RewP might relate to these different facets of perceived bonding in opposing ways. Mulligan et al.’s (2021) findings that RewP is associated with pleasure in proximity may also reflect the general links between the RewP in monetary reward tasks and the tendency to experience positive affect (Kujawa et al., 2015). In our study examining social RewP to infant cues specifically, mothers who are more responsive to potential images of their own infant may also be more sensitive to potential concerns about the relationship. Research is needed with more objective observational methods (e.g., recorded and coded free play tasks) to clarify whether alterations in RewP magnitude are related to behavioral indicators in the quality of mother–infant relationships or specifically maternal perceptions of these experiences (Edwards et al., 2021; Nascimento et al., 2023; Wittkowski et al., 2020). Future research is needed to examine the concurrent and prospective associations between RewP to monetary and social gains with perceived bonding.

We also sought to evaluate links between prenatal and postpartum depressive symptoms with perceived bonding difficulties. Consistent with previous findings, we found that prenatal depressive symptoms were associated with more bonding difficulties (Fransson et al., 2020; Garon-Bissonnette et al., 2024; Hare et al., 2021). We also found a small positive association, although it did not reach statistical significance, between bonding difficulties and PPD symptoms. Past research has linked maternal depressive symptoms in the postpartum period with maternal-reported difficulties in bonding (Letourneau et al., 2013; Moehler et al., 2006).

Furthermore, we sought to evaluate the bivariate associations among responsiveness to social reward, perceived bonding, and PPD symptoms. Contrary to our hypothesis, we observed that greater social RewP, rather than blunted social RewP, was associated with bonding difficulties. These findings contrast those of a recent study that found higher levels of RewP in the antenatal period to monetary gains were associated with greater maternal-reported pleasure in proximity to the infant during the postpartum period (Mulligan et al., 2021).

In addition, contrary to our hypothesis, we found no associations between social RewP and prenatal or postpartum depressive symptoms. Regarding bivariate correlations, there was a trending positive association between social RewP and postpartum depressive symptoms. These findings were surprising and may be driven in part by comorbid anxiety symptoms, such that new mothers high in anxiety may be more reactive to potential infant cues. Furthermore, these null findings align with prior literature that did not show significant bivariate, cross-sectional associations between RewP and depressive symptoms (Novak & Foti, 2015; Pegg et al., 2019). Although there are links between brain regions involved in reward processing and depression, the magnitude of effects is known to be relatively modest and some research in this domain has failed to yield significant main effects (Novak & Foti, 2015; Pegg et al., 2019). Our moderation findings are generally consistent with prior literature showing that low RewP may reflect vulnerability to depression in combination with other risk factors, such as exposure to stressful events (Pegg & Kujawa, 2024). For example, larger social Rewp may be associated with more worry or concern in mothers about the bond with their infant.

Similar to results from another study using a peer interaction social reward task in emerging adults, we did not find a significant bivariate correlation between social RewP and depressive symptoms (Pegg et al., 2021). However, similar to our findings, this study found a significant interaction between social RewP and a predictor related to reward sensitivity in relationship to depressive symptoms. These findings highlight the need to consider multiple factors in addressing the relationship between reward responsiveness and clinical neuroscience research across the lifespan.

Our analyses showed that prenatal depression was prospectively associated with perceived bonding difficulties. This finding follows a mixed body of literature regarding the directionality of the association between bonding difficulties and later depression. Greater perceived bonding difficulties is likely to be a source of stress that can increase risk for postpartum depressive symptoms (Hiraoka et al., 2024; Ohara et al., 2017). Conversely, preexisting depression is known to impact how experiences, such as those related to parenting, are processed and perceived (Harkness & Monroe, 2016). As such, it is quite possible that these associations are bidirectional at least when considering parents’ perceptions of bonding difficulties, but this association may present differently when considering observational assessments of bonding. For example, depressive symptoms in pregnancy may relate to perceptions but not necessarily observable indicators of bonding difficulties (Nath et al., 2019). Therefore, future research should integrate multiple methods to examine bonding difficulties and depressive symptoms during pregnancy to further discern the time course and directionally of associations between bonding difficulties, reward processing, and peripartum depressive symptoms.

A key strength of this study is the use of social rather than monetary reward responsiveness allowed us to build on previous findings linking reward sensitivity and maternal bonding (Mulligan et al., 2021). However, the present study has two key limitations that also warrant consideration. First, we relied on maternal-reported measures of difficulties in bonding that focused on participants’ perceptions. Although the PBQ is a validated questionnaire to assess maternal bonding, it would be useful to determine whether these patterns extend to the caregiving relationship as assessed via observational measures of interactions between caregivers and their infants. Second, the current study sample is relatively small, and replications in larger samples are needed.

## Conclusions

The current study examined whether EEG measures of social reward responsiveness in postpartum women may moderate the association between perceived bonding impairments and PPD. Our findings provide preliminary evidence that personally salient social reward may be a relevant measure of brain function in the context of maternal perceived bonding and PPD symptoms. Future research may evaluate whether reduced reward responsiveness assists in the early detection of risk for postpartum depression. If so, interventions used to increase neural reward responsiveness may be useful for supporting both bonding and mental health outcomes for women across the peripartum period. This can lead to additional future research testing the impact of parent-infant bonding behavioral interventions on social RewP and PPD symptoms.

## Data Availability

Data or materials for the experiments are available upon request, and none of the experiments was preregistered.

## Footnotes

1 Because reaction time data were missing for four participants due to technical error, 87 participants were included in the reaction time analyses. Reaction time shorter than 100 ms or longer than 2000 ms on individual trials were excluded as outliers. Averages were calculated separately for each condition (i.e., incentive, nonincentive).

## Code availability

Upon request to the corresponding author.
